# Prepartum Dietary Supplementation of Potassium Humate Improves Postpartum Lactation Performance, Metabolic Profile of Multiparous Cows, and Immune Response of Their Calves

**DOI:** 10.3390/ani15020279

**Published:** 2025-01-20

**Authors:** Cangir Uyarlar, Abdur Rahman, Eyup Eren Gultepe, İbrahim Sadi Cetingul, Muhammad Uzair Akhtar, Ismail Bayram

**Affiliations:** 1 Department of Animal Nutrition, Faculty of Veterinary Medicine, Afyon Kocatepe University, ANS Campus, Afyonkarahisar 03200, Türkiye; cangiruyarlar@hotmail.com (C.U.); eegultepe@gmail.com (E.E.G.); sadicet@yahoo.com (İ.S.C.); ibayram@aku.edu.tr (I.B.); 2 Department of Animal Sciences, University of Veterinary and Animal Sciences, Lahore, Jhang Campus, Jhang 35200, Pakistan; 3 Department of Animal Nutrition, Cholistan University of Veterinary and Animal Sciences, Bahawalpur 63100, Pakistan

**Keywords:** periparturient cow, metabolic health, dry period, milk yield, immunity, calf

## Abstract

This research was carried out to determine the effects of potassium humate on the lactation performance and metabolic parameters of dairy cows during the transition period. Potassium humate was added to the concentrate feed at the following levels: (a) control (0%), (b) 0.5%, (c) 1%, (d) 1.5%, and (e) 2% humas, during the dry period from −60 to 0 days until calving. The results indicated that the total milk yield after 305 days was higher in the 0.5% group than in the 2% humic acid group. The average daily milk yield from lactation was also greater in the 0.5% group than in the 2% humic acid group. In terms of metabolic health and blood biochemistry, lymphocytes, neutrophils, monocytes, NEFAs, and BHBA were different among the treatment groups. No effects were detected on the blood physiology parameters of the calves. The IgG concentration in the colostrum and serum of calves on day 1 and 2 were higher in the 0.5% and 1% humic acid groups, respectively, than in the other groups. Overall, adding humic acid, especially at the dose of 0.5%, to the concentrate feed of dairy cows during the dry period resulted in an increased postpartum milk yield for the cows and increased serum IgG in both the cows and calves, with decreased NEFAs on the calving day and decreased postpartum BHBA for cows.

## Simple Summary

This study evaluated the effects of humic acid supplementation during the dry period on the postpartum lactation performance and metabolic health of transition dairy cows. The results indicated that 0.5–1% humic acid supplementation to concentrate feed during the dry period had positive effects on the subsequent lactation performance by improving milk yield and metabolic health in terms of reduced β-hydroxybutyrate and non-esterified free fatty acids.

## 1. Introduction

The most challenging period in feeding dairy cows is the transition period, which includes the last three weeks of pregnancy and the first three weeks of subsequent lactation [1]. On dairy farms, 30–50% of cows experience health problems during this stage and nearly 75% of metabolic and infectious disease occurrences happen during this period [2,3]. It is partially associated with a negative energy balance, leading to excessive fatty acid inclusion in the leukocytes due to the mobilization of adipose tissues, which contributes to reduced postpartum lactation performances for dairy cows [4]. During transition, lymphocyte and neutrophil functions are also impaired [5], which ultimately contributes to impaired postpartum milk production and leaves the cows highly susceptible to diseases during the transition period [6,7]. Improved postpartum lactation performance and low disease incidence in transition cows can be achieved through different nutritional strategies. Supportive supplements are one of the best options for dairy cows to enable a successful transition period. For this purpose, various feed additives, boluses, or injectable supplements are used [8].

Humates comprise humic, fulvic, and ulmic acids and some microminerals from humus. Humic substances are hydrophilic and have a high molecular weight; compounds of humus [9] also demonstrate beneficial properties such as anti-microbial effects [10], anti-oxidant effects [11], anti-inflammatory effects [12], anti-radical effects [13], being an immune system regulator [14], and being a blood sugar regulator [15]. Humic acid can stimulate the metabolic process in the cell membrane by increasing oxidative processes, resulting in improved nutrient absorption. This increased nutrient absorption, combined with efficient rumen fermentation, may result in improved lactation performances for the dairy cows. The primary modes of action through which humic acid has beneficial effects include the following: (1) an increased number of immune receptors in the gastrointestinal tract, enabling protection from pathogens; (2) making defensive layers over epithelial mucosa for protection from infectious agents; (3) changes in the microbial population to reduce metabolic needs and enable the increased availability of nutrients, especially proteins and carbohydrates; and (4) a detoxification function in the gut [16]. In the literature, humic acid substances have been shown to improve milk yields, protein digestibility, and volatile fatty acid levels in goats, in addition to increased blood glucose levels, indicating its potential to improve energy balance and health status and to prevent metabolic disorders [17]. Cows fed humic acid demonstrated faster growth [18] and an increased milk yield [19,20]. Previous reports on blood physiological profiles indicated that humic acid can increase lymphocytes and the production of glycoproteins, which regulate the immune system [21]. Moreover, the liver is the most important organ for a smooth transition period in dairy cows, from the non-lactating to the lactating phase. Despite the observed beneficial effects of humic acid on liver and rumen fermentation [20], health, digestion, immunity [22,23], nitrogen binding capacity [24], and neutrophil activity [25], the influence of humic acid on lactation performance and the metabolic profile of transition cows remain unclear. Its anti-oxidant properties, combined with reduced histopathological changes in the liver and kidney, indicate humic acid’s synergistic effect [26] and its potential utilization during the transition period of dairy cows.

Despite the above-mentioned beneficial effects of humic acid, no study has been conducted, as per the authors’ knowledge, to observe the effects of humic acid supplementation on transition dairy cows. In the present study, we hypothesized that humic acid supplementation during the dry period would improve the postpartum lactation performance, with positive effects on the physiological adaptations around calving. Therefore, this study was carried out to examine the effects of varying humic acid (potassium humate) levels in concentrate feed during the dry period on the postpartum lactation performance of Holstein dairy cows and to reveal the effects of humic acid on the physiological status and immunity of both the dam and calf.

## 2. Materials and Methods

### 2.1. Cows

This study was carried out at a Holstein dairy farm of Niğtaş A.Ş. Agriculture and Animal Husbandry, with a total of 3000 Holstein cows, in Niğde, Central Anatolia, Türkiye, at 37.9698° N and 34.6766° E. All the experimental procedures were approved by the animal experiment ethics committee of Afyon Kocatepe University (reference number AKÜHADYEK-108-22 number 125 dated 30-11-2022). The experiment began with the initiation of the dry period (−60 d until calving) and dietary treatments were continued until the day of calving. A total of 75 multiparous Holstein dairy cows with similar parity (3rd) entered the dry period on the 220th day of their pregnancy and were used in this study. All enrolled cows started their dry periods within five days of the start of the experiment. The cows were divided into five treatment groups according to their body weight, body condition scores, and previous lactation milk yields to balance the treatment groups. During the last days of lactation, because of a decreased milk yield, the cows were fed a diet with a high forage-to-concentrate ratio. Consequently, changes in the dry period plane of nutrition did not pose any considerable concerns.

### 2.2. Experimental Design and Treatments

This study was carried out using a completely randomized design. The treatment groups included the following: (1) control, no supplement; (2) 0.5% humas, 0.5% humic acid in the concentrate feed; (3) 1% humas, 1% humic acid in the concentrate feed; (4) 1.5% humas, 1.5% humic acid in the concentrate feed; and (5) 2% humas, 2% humic acid in the concentrate feed. Potassium humate, which is a mixture of certain amounts of leonardite and potassium hydroxide, was used as a humic acid in this study. Cows were fed total mixed rations (TMRs). As seventy-five enrolled cows were divided into five groups, these groups were further divided into subgroups of five cows each, and feed intake data were collected on the basis of these subgroups (three subgroups for each treatment group) during the prepartum period and individually after birth. However, after the experimental period, all cows were fed the same lactating diet ad libitum throughout the lactation period under similar management conditions. Potassium humate was obtained from Türkiye Coal Enterprises (Ankara, Türkiye) and added to the concentrate at the mixing stage to prepare five different concentrates, according to the treatment levels of this study, at a commercial feed mill (Kocayusuf Feed Mill, Adana, Türkiye). The humic acid contained 19.1, 3.46, 10.4, and 8.65% crude protein, crude fat, crude ash, and cellulose contents, respectively, as reported previously [27]. The concentrates with different levels of humic acid were mixed with forage portions, in order to feed TMR-based diets to cows according to their treatment groups. The animals were fed twice daily, at 06:00 and 18:00 h. The TMRs for all groups were prepared with the same calorie and nitrogen contents. The average crude protein and metabolizable energy contents of the concentrate were 21.7% of dry matter (DM) and 2.34 Mcal/kg of DM, while the concentrate intake was 1.5 and 3.5 kg during the far-off and close-up periods, respectively. The average postpartum DM intake (DMI) was 21 kg/day per cow. The crude protein and metabolizable energy contents of the lactating concentrate were 21.3% and 2.54 Mcal/kg of DM, respectively. The concentrate feed and nutrient composition of the TMRs are presented in Table 1 and Table 2, respectively.

### 2.3. Sample Collection and Analysis

The feed was weighed and given to the treatment groups daily to determine the feed consumption. Samples were taken from all raw materials used at the beginning of the experiment and these values were used to formulate the rations. The TMR samples were collected weekly and frozen at −20 °C until analysis. Feed intake data were collected three days per week during the prepartum period on a subgroup basis. On calving day and during the postpartum period, feed intake data were collected individually for each cow on a daily basis. At the end of the experimental phase of this study, all TMR samples were mixed and made homogeneous. The samples were analyzed for dry matter, crude ash, crude protein, crude fiber, and crude fat contents [28], while ADF and NDF analyses were conducted following the methodology of Van Soest et al. [29].

The daily milk yield of each cow was recorded individually. Cows were milked thrice daily using the herd management system Dairy Plan C21 (GEA Farm Technologies GmBH, Bönen, Germany). The body condition scores of all cows were measured on days −60, −30, 0, 15, and 60 relative to calving, following the methodology of Edmonson et al. [30]. Blood samples from all cows were collected from the coccygeal blood vessels on days −60, −53, −46, −39, −32, −25, −18, −11, −4, 0, 1, and 2 relative to calving. The blood samples were collected in evacuated tubes containing serum separator gel, and three tubes were collected from each cow. Serum and plasma were obtained by centrifuging the blood at 2000× *g* for 10 min. Serum and plasma samples were stored at −20 °C until analyzed. Blood was taken from all newborn calves on days 0, 1, and 2 following birth. Calves were not allowed to suckle their dams and colostrum samples were collected from all animals immediately after parturition (within 45 min) and on the first and second days postpartum. These samples were then stored at −30 °C until analysis was conducted. In the whole blood samples taken in this study, the total leukocyte count (TLS), lymphocyte count, monocyte count, hemoglobin amount, hematocrit percentage, MCV (mean erythrocyte volume), MCH (mean corpuscular hemoglobin), MCHC (mean corpuscular hemoglobin concentration), PLT (platelet) amount, and MPV (mean platelet volume) were analyzed using the Mindray BC 2800 device (Mindray Medical International Ltd., Shenzhen, China). In serum and plasma samples, blood parameters related to metabolism and immunity, NEFAs (non-esterified fatty acids), BHBA (beta-hydroxybutyric acid), glucose, total cholesterol, triglyceride, ALT (alanine aminotransferase), AST (aspartate aminotransferase), ALP (alkaline phosphatase), GGT (gamma glutamyl transferase), and total immunoglobulin G levels were determined using commercially available kits and the Chemwell 2910 fully automatic ELISA reader (Awareness Technology Inc., Palm City, FL, USA).

### 2.4. Statistical Analysis

Data collected on a daily basis were aggregated into weekly means before statistical analysis was conducted. Prepartum, postpartum, and calving day data were analyzed separately, as reported previously [31,32]. Experimental data were analyzed using the GLIMMIX procedure in SAS (SAS On-Demand for Academics; SAS Institute Inc., Cary, NC). A repeated measures analysis was conducted for variables measured over time; week or day was considered as a repeated measure. Our statistical model included the fixed effects of dietary humic acid level as the treatment, the effect of time measured as week or day, and the interaction between treatment and time. The random error term used for all the models was the cows within each humic acid treatment level, and a covariance structure yielding the lowest Akaike’s information criterion was used [33]. Following this methodology, an autoregressive covariance structure was the best fit for data in this study. The Satterthwaite option was used to calculate the degree of freedom. The subgroup was used as an experimental unit for feed intake data. For the parameters not measured over time, the GLIMMIX procedure in SAS was used, without a repeated measures statement. The statements of linear and quadratic polynomial contrasts were included in the analysis of all parameters evaluated to observe the dose–response effects of increasing potassium humate levels. The results are presented as least-square means with standard error of the means. Statistical significance was set at *p* < 0.05 using Tukey’s multiple comparison test.

## 3. Results

The effects of humic acid supplementation in dairy cows during the dry period on subsequent lactation performance and DMI are presented in Table 3. The total milk yield over 305 days of lactation and average milk yield over the lactation period increased linearly (*p* < 0.01) with a 0.5% humic acid supplementation level, while these measures decreased with a 2% humic acid supplementation level (*p* < 0.01), in a quadratic fashion (*p* < 0.01). The total milk yield and mean milk yield during complete lactation days were affected by dietary treatments (*p* < 0.05), as an increase was observed in the 0.5% humic acid group, while a linear decrease was observed in the 2% humic acid group (*p* < 0.05), irrespective of the total number of complete lactation days (*p* > 0.05). A linear decrease (*p* < 0.05) in the peak milk yield was observed in the 2% humic acid group compared with in the control, 0.5, and 1% humic acid groups (*p* < 0.01). Colostrum IgG content also responded to dietary changes (*p* < 0.05), as it increased with a 0.5% humic acid level in a quadratic fashion (*p* < 0.05). The intake of DM during the prepartum period linearly increased (*p* < 0.05) in the 0.5, 1, and 1.5% humic acid groups compared with in the control and 2% humic acid treatment groups (*p* < 0.05). The DMI during the postpartum period linearly increased (*p* < 0.05) in the 0.5 and 1% humic acid groups compared with in the other groups.

During the post-experiment lactation period, the 0.5% and 1% humic acid groups had lower disease incidence rates than those of the other groups (Table 4).

The effects of humic acid supplementation on serum physiological parameters during the pre- and postpartum periods are presented in Table 5. During the prepartum period, the TLS of cows increased linearly (*p* < 0.05) in the 0.5 and 1% humic acid groups, compared with in the 2% humic acid group (*p* < 0.05). The lymphocyte concentration increased linearly (*p* < 0.01) in the 0.5 and 1% humic acid groups compared with in the control group (*p* < 0.05). A linear effect (*p* < 0.05) was observed for the monocyte content, as this measure increased in cows fed 1% humic acid, compared with those fed 1.5% humic acid (*p* < 0.05). The platelet count was higher in the control and 2% humic acid groups than in cows in other groups (*p* < 0.05). Neutrophils, RBCs, hemoglobin, hematocrit, the MCV, MCH, the MCHC, and the MPV remained unaffected by different dietary humic acid levels during the prepartum period (*p* > 0.05). On the calving day, a linear effect (*p* < 0.05) was observed for the TLS, as it increased in the 1% humic acid group, compared with in the 2% humic acid group (*p* < 0.05). The monocyte count was higher in the control and 0.5% humic acid groups than in the 2% humic acid group (*p* < 0.05). The MCHC increased in cows in the 2% humic acid group, compared with those in the 0.5 and 1.5% humic acid groups, during the dry period (*p* < 0.05). Lymphocytes, neutrophils, RBCs, hemoglobin, hematocrit, the MCV, MCH, platelets, and the MPV of cows on calving day (day 0) remained unaffected by prepartum dietary humic acid levels (*p* > 0.05). During the postpartum period, lymphocyte contents decreased linearly in the control group compared with in the other groups (*p* < 0.05). The monocyte count decreased linearly (*p* < 0.05) in the 2% humic acid group in a quadratic fashion (*p* < 0.05) compared with in the control, 0.5, and 1% humic acid groups (*p* < 0.05). The postpartum MPV content increased linearly (*p* < 0.05) with a quadratic effect (*p* < 0.05) in cows fed 0.5% humic acid, compared with in those fed 2% humic acid (*p* < 0.05). The blood TLC, lymphocytes, neutrophils, RBCs, hemoglobin, hematocrit, MCV, MCH, MCHC, and platelet count of cows during the postpartum period remained unaffected by prepartum dietary humic acid levels (*p* > 0.05). The concentrations of the TLC, lymphocytes, neutrophils, monocytes, and platelets during the prepartum period and of RBCs during the postpartum period changed over time in all cows (*p* < 0.05). An interaction between treatment and time was also observed for the prepartum TLC, lymphocytes, neutrophils, and monocytes, and postpartum neutrophils (*p* < 0.05).

The effects of humic acid supplementation on the serum biochemical parameters of cows during the pre- and postpartum periods are presented in Table 6. During the prepartum period, serum NEFA contents decreased linearly with increasing humic acid supplementation (*p* < 0.05). Serum IgG concentration increased linearly (*p* < 0.05) in cows fed with 0.5 and 1% humic acid, compared with in cows in the control and 2% humic acid groups (*p* < 0.05). Serum glucose, ALT, AST, ALP, GGT, triglyceride, cholesterol, and BHBA were not affected by prepartum dietary treatments (*p* > 0.05). On calving day, the serum NEFA content decreased linearly (*p* < 0.05), whereas the serum IgG content increased in the 0.5 and 1% humic acid groups compared with in the control and 2% humic acid groups (*p* < 0.05). No changes were observed in the glucose, ALT, AST, ALP, GGT, triglyceride, cholesterol, and BHBA levels of cows on calving day (*p* > 0.05). During the postpartum period, higher triglyceride contents were observed in the 2% humic acid group, compared with in the 1.5% humic acid group (*p* < 0.05). The serum BHBA content decreased linearly (*p* < 0.05) in the 0.5 and 1% humic acid groups, compared with in the other groups. A quadratic effect (*p* < 0.05) indicated that the concentration of serum IgG was higher in cows in the 0.5% humic acid and control groups, compared with in those on the 2% humic acid diet (*p* < 0.05). All biochemical parameters changed over time during the prepartum period (*p* < 0.05) but not during the postpartum period (*p* > 0.05). No interaction between treatment and time was observed for any biochemical parameter, except prepartum NEFAs and IgG and postpartum triglyceride contents (*p* < 0.05).

The effects of humic acid supplementation on the serum physiological parameters of calves are presented in Table 7. On calving day, the serum TLC, lymphocytes, neutrophils, monocytes, RBCs, hemoglobin, hematocrit, MCV, MCH, MCHC, platelets, MPV, and IgG concentrations were similar in all treatment groups (*p* > 0.05). The serum cholesterol content was higher on calving day in the 2% humic acid group than in the 1.5% humic acid group (*p* < 0.05). On day 1, the monocyte content increased linearly (*p* < 0.05) with increasing levels of humic acid, but it did not increase in the control group. The MCV was affected by the dietary treatments, in linear as well as quadratic fashion (*p* < 0.05), as an increased MCV was observed in the 0.5% humic acid group, compared with in the 1.5% humic acid group (*p* < 0.05). The highest MCH and MPV contents were observed in the control group, as a quadratic effect, compared with in the 1.5% humic acid group (*p* < 0.05). The cholesterol content was highest in the 2% and lowest in the 1.5% humic acid group, compared with in the other groups (*p* < 0.05), as a quadratic effect (*p* < 0.05). The serum IgG levels in the 0.5% humic acid group were higher than in the control group, but they linearly decreased in the 2% humic acid group on day 1 after calving (*p* < 0.05). On day 2 after calving, the cholesterol content quadratically increased in the 2% humic acid group, whereas the lowest values were observed in the 1.5% humic acid group, compared with in the other groups (*p* < 0.05). The serum IgG levels were higher in the 1% humic acid group, compared with in the control, 1.5%, and 2% humic acid groups (*p* < 0.05).

The effects of humic acid supplementation on the serum biochemical parameters of calves are presented in Table 8. Serum ALT, AST, ALP, and GGT concentrations in calves remained unaffected by the supplementation of humic acid to cows during the dry period (*p* > 0.05).

The effects of humic acid supplementation on the BCS of dairy cows are presented in Table 9. The supplementation of humic acid during the dry period had no effect on the BCS of the cows on days −60, −30, 0, 15, and 60 relative to calving (*p* > 0.05).

## 4. Discussion

This study revealed the positive effects of humic acid supplementation on milk yield, in terms of 305-day total milk yield, 305-day mean milk yield, and peak milk yield. These results indicated that supplementing dairy cattle with humic acid during the dry period had a dose-dependent effect on milk yield throughout the subsequent lactation period. The effect was positive in terms of milk production at a dose of 0.5% but negative at the dose of 2%. Limited studies are available on the impacts of humic acid supplementation, especially on productivity parameters, in dairy cows. However, consistent with the results of this study, Griban [34] found that providing humic acid to dairy cows increased their milk yield. This could be attributed to their improved postpartum DMI and improved metabolic health, which are considered the primary regulators of milk production. Additionally, the positive effects of humic acid on rumen fermentation, nutrient absorption, and glucose-sparing effects have been reported previously [17]. Humic acid can reduce endotoxin and mycotoxin absorption, as well as the growth of pathogenic bacteria [35]. Rumen microbes can use humic acid as an electron receptor to trigger bacterial growth and to utilize nitrogen [36]. Moreover, it not only reacts with biologically active substances but also causes a decrease in the rumen protozoal population, which alters rumen fermentation and the rumen pH [35]. All these effects may have contributed to the improved nutrient absorption and lactation performance observed with humic acid supplementation, particularly at the levels of 0.5% and 1% in this study. Keeping in mind the effects of dry period treatment on postpartum lactation throughout the subsequent lactation period, the increase in lactation of 3.78% following the 0.5% humic acid treatment, compared with the control group, was interesting. However, the lactation reduction observed for the 2% humic acid group could be attributed to various factors, including the discovery of an optimum level, the presence of any other compounds in humic acid which are not beneficial, or the interaction of humic acid with other nutrients. Previously, humic acid supplementation has been reported to inhibit the protozoal population and to increase, decrease, or have no effect on rumen pH [17,35,36]; these varying results indicate our limited understanding of humic acid’s effects on the rumen at various dose levels. Although the negative effects of a 2% level of humic acid might be partially associated with the observed reduced DMI, compared with other levels of humic acid, inconsistent findings are reported in the literature regarding the effects of different supplementation levels [37,38]. Nevertheless, in this study, these differences are primarily between the 0.5 and 2% humic acid groups but do not occur in the control group for most of the parameters evaluated.

It is inevitable that cows develop immunosuppression due to the metabolic and immunological stress they experience during the periparturient period. This period is critical for dam and calf health [39,40], as both become vulnerable to various infectious diseases during this time [41]. In this study, significant differences were found between the groups in terms of RBC, WBC, lymphocyte, neutrophil, and monocyte values, which are blood physiology parameters, in both the prenatal and postnatal periods. It has previously been observed that the total blood leukocyte count increases rapidly on and after the day of parturition [41]. Merrill et al. [42] found a significant increase in lymphocyte and eosinophil numbers and a decrease in neutrophils up to ten days after parturition, with no change observed in the number of monocytes. Some researchers [43] reported that the average blood total leukocyte, lymphocyte, and neutrophil counts decrease rapidly after parturition but gradually increase until the 20th day of lactation. The same researchers reported that the prenatal red blood cell count is higher than the postnatal red blood cell count. However, these changes were not found in the current study. The reasons for this may include the sheltered conditions where the animals were housed, the smaller and more controlled numbers of animals in this experiment, the care taken in meeting daily nutritional needs by feeding the cows TMRs, and the significant reduction in environmental stress factors during the birth period. In addition, changes over time occurred as expected, and all values remained within the reference ranges. The number of studies examining the hematological parameters and factors affecting them in calves is limited. Studies have generally focused on comparing calves and adult cattle [44]. In the current study, no statistically significant difference was observed in blood physiology parameters.

The levels of IgG in the colostrum were significantly statistically different between the groups. Consistent with the blood data, the colostrum IgG level in the 0.5% humic acid group was found to be statistically higher than in all other groups. The total amount of IgG in the blood is one of the most important parameters indicating an animal’s immune status. It was observed in the current study that humic acid, especially at levels of 0.5% and 1%, increased the immune response of dairy cows during the parturition period and increased the level of IgG, which was reflected in the blood. Humic acid is an important source of various nutrients that stimulate oxygen transport and immunity in various animal species [45], which could explain the increase in IgG contents observed in this study. However, our understanding of the exact mechanisms involved in stimulating this immunity is limited [46]. A few studies have examined the effects of various feeding practices for dams applied before parturition on the health and development of newborn calves. Studies have mostly focused on periods of intense stress, such as weaning or transportation [47,48,49], because immunosuppression develops with increased cortisol release during stress [27]. In the presented study, adding different levels of humic acid to the feed of dairy cows before parturition did not cause a significant difference in the blood physiology parameters of their calves. However, blood IgG levels in calves were higher in the 0.5% and 1% humic acid groups than in the other groups, which can be explained by the total IgG levels in the blood and colostrum of cows during the postpartum period. Limited data are available on changes in calf serum profiles due to dietary supplements during the prepartum period. However, the positive effects of humic acid on calf serum and liver might be attributed to the increased serum IgG contents of calves.

Although few differences were observed in blood biochemical parameters between the treatment groups, considerable changes were noted in NEFA and BHBA levels, with lower values in the 0.5% and 1% humic acid groups. Serum AST, GGT, and ALT concentrations indicate hepatic functions in farm animals [50]. Sevınc et al. [51] reported a connection between the serum AST and GGT levels of liver enzymes and fatty livers. Accordingly, there is a positive relationship between increases in serum AST levels and the level of liver fat. As the AST level increases, the rate of fat accumulation in the liver increases. However, humic acid substances, sources, preparations, animal species, extraction techniques, dosage, and dietary nutrients can cause variations in response.

The concentrations of NEFAs and BHBA are parameters that provide important information about metabolic diseases, especially fatty liver and ketosis, during the transition period. High levels of both parameters in the blood indicate that the animal is highly susceptible to metabolic diseases [52,53,54]. In particular, a rapid increase in NEFAs around calving is linked to the initiation of TG infiltration [55,56]. In this study, low levels of NEFAs and BHBA in the 0.5% and 1% humic acid groups suggested that adding humic acid to the diets of dairy cows before birth helps to maintain NEFA and BHBA levels, which are indicators of energy metabolism being within the normal range. In each case, as indicated by Musco et al. [57], the levels of NEFAs demonstrated that the nutritive requirements of dairy cows were satisfied for each group, as supported by the BCS of the cows. Additionally, significant differences within the groups were observed for changes over time. As per the authors’ knowledge, this is the first study to report the positive effects of prepartum humic acid supplementation in the maternal diet not only on postpartum lactation performance but also on blood biochemistry parameters in newborn calves. The results of this study indicate the promising effects of humic acid supplementation during the prepartum period, which warrants further investigation regarding the dose level, changes in milk composition, and duration of supplementation.

## 5. Conclusions

The findings of this study indicate that adding humic acid at 0.5% and 1% levels during the dry period improved the postpartum lactation yield and average milk production of dairy cows. Moreover, the biochemical and metabolic profiles of the periparturient cows were also improved with humic acid supplementation, specifically through decreasing the NEFA and BHBA concentrations. These positive effects were reflected in an increased milk yield throughout the subsequent lactation period. However, when 2% humic acid was added to the diet during the dry period, it negatively impacted the blood biochemistry and milk yield parameters of the cows. Overall, this study indicates that adding 0.5–1% humic acid to their diet during the dry period increased the postpartum milk yield and improved the metabolic health of dairy cows.

## Figures and Tables

**Table 1:** Composition of the concentrates.

	Treatment group					
Composition, g/kg	Control	0.5% Humas	1% Humas	1.5% Humas	2% Humas	Lactating Concentrate
Wheat	160	154	148	142	136	200
Barley	120	120	120	120	120	250
Molasses	60.0	61.0	62.0	63.0	64.0	50.0
Canola meal	186	186	186	186	186	40.0
Corn gluten 60%	30.0	30.0	30.0	30.0	30.0	50.0
Soybean meal	100	100	100	100	100	130
Wheat bran	120	120	120	120	120	100
Corn DDGS, dry	150	150	150	150	150	125
Magnesium sulfate	70.0	70.0	70.0	70.0	70.0	-
Premix	2.00	2.00	2.00	2.00	2.00	20.0
Salt	-	-	-	-	-	10.0
Limestone	-	-	-	-	-	20.0
Toxin binder	2.00	2.00	2.00	2.00	2.00	5.00
Humic acid	-	5.00	10.0	15.0	20.0	-

**Table 2:** Ingredients and nutrient composition of total mixed rations (% of DM).

Item	Far-Off Dry Period	Close-Up Dry Period	Lactation
Corn silage	14.3	14.5	34.0
Wheat straw	55.2	39.3	
Alfalfa hay	9.69	9.67	22.0
Brewer’s grains, wet	1.75	1.72	3.02
Sugar beet pulp, wet	1.35	1.33	2.35
Orange pulp, wet	1.13	1.15	2.17
Pomegranate pulp, wet	1.49	1.51	2.53
Concentrate feed	15.1	30.8	36.9
Nutrients			
Crude protein, % DM	10.8	12.9	15.9
ME, Mcal/kg of DM	17.5	19.2	2.51
Ca, % DM	0.54	0.53	0.76
P, % DM	0.18	0.19	0.34

**Table 3:** Effects of humic acid supplementation ^1^ during dry period on subsequent lactation parameters of dairy cows.

Item	Groups					SEM	*p*-Values		
	Ctrl	0.5% Humic Acid	1% Humic Acid	1.5% Humic Acid	2% Humic Acid		Treat	Linear	Quadratic
Total milk yield (305 days), kg	9012 ^ab^	9353 ^a^	8957 ^ab^	8813 ^ab^	8365 ^b^	159.8	0.001	<0.001	0.030
Mean milk yield (over 305 days), kg/d	29.5 ^ab^	30.7 ^a^	29.4 ^ab^	28.9 ^ab^	27.4 ^b^	0.524	0.001	<0.001	0.030
Total milk yield (during days in milk), kg	10,338 ^ab^	10557 ^a^	10,228 ^ab^	10,101 ^ab^	9742 ^b^	168.7	0.018	0.003	0.135
Days in milk	405	401	404	403	403	2.776	0.815	0.791	0.613
Mean milk yield (during days in milk), kg/d	25.5 ^ab^	26.4 ^a^	25.4 ^ab^	25.1 ^ab^	24.2 ^b^	0.433	0.016	0.005	0.098
Peak milk yield, kg/d	46.7 ^a^	46.3 ^a^	46.1 ^a^	45.6 ^ab^	42.3 ^b^	0.868	0.003	<0.001	0.066
Colostrum IgG, mg/dL	62.6	69.1	65.5	64.8	61.2	1.662	0.014	0.173	0.007
DMI-BP, kg/d ^2^	12.6 ^b^	13.0 ^a^	12.9 ^a^	13.0 ^a^	12.5 ^b^	0.067	<0.0001	<0.001	0.555
DMI-AP, kg/d ^2^	10.2 ^b^	10.7 ^a^	10.6 ^a^	10.3 ^b^	10.4 ^b^	0.062	<0.0001	<0.001	0.265

^1^ Ctrl: without supplement; 0.5% humic acid; 1% humic acid; 1.5% humic acid; 2% humic acid. ^2^ DMI-BP: dry matter intake—prepartum (−60–1 days); DMI-AP: dry matter intake—postpartum (0–2 days). ^a,b^ Values with different superscripts in the same row differ significantly (*p* ≤ 0.05).

**Table 4:** Health status of (number of cows in) each group during the whole lactation period (after the experiment).

Groups	Retention of Placenta	Metritis	Mastitis	Ketosis	Abomasum Displacement	Cystic Ovarian	Inactive Ovarian	Culled
Control	2	5	9	5	-	12	3	5
0.5% humic acid	1	2	4	3	-	4	1	2
1% humic acid	1	3	5	2	-	6	1	2
1.5% humic acid	3	4	7	5	-	11	2	4
2% humic acid	4	5	10	7	-	12	3	4

Control: without supplement; 0.5% humic acid; 1% humic acid; 1.5% humic acid; 2% humic acid.

**Table 5:** Effects of humic acid supplementation ^1^ on serum physiological parameters of cows during the pre- and postpartum periods ^2^ and on calving day.

Item ^3^	Treatment					SEM	*p*-Values				
	Control	0.5% Humic Acid	1% Humic Acid	1.5% Humic Acid	2% Humic Acid		Treat	Linear	Quadratic	Time	Treat × Time
Prepartum											
TLC, 10^9^/ml	6.92 ^ab^	7.04 ^a^	7.05 ^a^	6.91 ^ab^	6.80 ^b^	0.053	0.009	0.006	0.241	<0.001	<0.001
Lymphocytes, %	2.91 ^b^	3.11 ^a^	3.16 ^a^	3.08 ^ab^	3.01 ^ab^	0.049	0.004	<0.001	0.117	<0.001	<0.001
Neutrophil, %	2.73	2.74	2.81	2.66	2.68	0.048	0.229	0.390	0.466	0.005	<0.001
Monocytes, %	0.33 ^ab^	0.34 ^ab^	0.34 ^a^	0.33 ^b^	0.34 ^ab^	0.001	0.021	0.046	0.450	<0.001	<0.001
RBC, 10^6^/dL	6.44	6.49	6.53	6.47	6.53	0.027	0.096	0.319	0.129	0.590	0.548
Hemoglobin, g/dL	10.2	10.5	10.5	10.4	10.6	0.076	0.062	0.205	0.146	0.570	0.864
Hematocrit, %	30.0	30.0	29.9	29.9	30.1	0.115	0.925	0.565	0.896	0.929	0.661
MCV, fL	46.9	47.1	47.1	47.0	47.0	0.050	0.476	0.075	0.712	0.541	0.877
MCH, pg	17.5	17.4	17.4	17.5	17.4	0.086	0.973	0.895	0.761	0.385	0.939
MCHC, g/dL	34.9	35.0	35.1	34.9	35.1	0.101	0.646	0.627	0.432	0.074	0.760
Platelets, 10/L	201 ^a^	201 ^ab^	199 ^b^	200 ^ab^	201 ^a^	0.365	0.015	0.094	0.199	<0.001	0.305
MPV, fL	5.44	5.45	5.46	5.45	5.44	0.005	0.268	0.102	0.357	0.327	0.278
Calving day											
TLC, 10^9^/ml	8.98 ^ab^	9.65 ^ab^	9.81 ^a^	9.61 ^ab^	8.73 ^b^	0.267	0.021	0.005	0.610	-	-
Lymphocytes, %	3.54	3.87	3.88	3.56	3.84	0.197	0.543	0.260	0.989	-	-
Neutrophil, %	3.14	2.88	2.77	2.76	2.79	0.142	0.297	0.218	0.081	-	-
Monocytes, %	0.36 ^a^	0.35 ^a^	0.34 ^ab^	0.34 ^ab^	0.33 ^b^	0.004	<0.001	0.784	<0.001	-	-
RBC, 10^6^/dL	6.27	6.19	6.29	6.24	6.20	0.037	0.270	0.666	0.680	-	-
Hemoglobin, g/dL	9.67	9.67	9.71	9.62	9.54	0.123	0.891	0.679	0.705	-	-
Hematocrit, %	31.0	31.0	30.9	30.9	31.1	0.157	0.942	0.844	0.722	-	-
MCV, fL	35.9	36.0	36.3	35.8	35.5	0.271	0.414	0.231	0.853	-	-
MCH, pg	11.4	11.6	11.6	11.9	11.4	0.224	0.639	0.624	0.389	-	-
MCHC, g/dL	34.6 ^ab^	34.2 ^b^	34.7 ^ab^	34.2 ^b^	35.1 ^a^	0.225	0.048	0.147	0.484	-	-
Platelets, 10/L	243	246	244	250	242	2.205	0.089	0.275	0.310	-	-
MPV, fL	5.47	5.41	5.46	5.43	5.45	0.015	0.123	0.060	0.858	-	-
Postpartum											
TLC, 10^9^/ml	9.05	9.25	9.23	9.13	8.94	0.196	0.792	0.277	0.831	0.787	0.394
Lymphocytes, %	3.57	3.86	4.07	3.80	3.75	0.133	0.130	0.036	0.258	0.215	0.570
Neutrophil, %	3.13	2.93	3.07	2.92	2.78	0.104	0.139	0.730	0.277	0.595	0.012
Monocytes, %	0.36 ^a^	0.36 ^a^	0.35 ^a^	0.34 ^ab^	0.33 ^b^	0.002	<0.001	0.008	<0.001	0.35	0.175
RBC, 10^6^/dL	6.28	6.34	6.30	6.35	6.40	0.042	0.308	0.770	0.316	<0.001	0.815
Hemoglobin, g/dL	9.69	9.84	9.85	9.67	9.70	0.099	0.564	0.159	0.652	0.371	0.719
Hematocrit, %	30.5	30.5	30.5	30.4	30.2	0.145	0.757	0.529	0.518	0.773	0.418
MCV, fL	34.2	34.4	34.3	34.0	34.3	0.154	0.528	0.422	0.506	0.511	0.936
MCH, pg	11.9	11.7	11.7	11.4	11.8	0.172	0.292	0.206	0.153	0.173	0.574
MCHC, g/dL	34.3	34.6	34.5	34.3	34.5	0.167	0.729	0.334	0.802	0.084	0.419
Platelets, 10/L	243	245	245	244	248	1.650	0.472	0.883	0.353	0.246	0.146
MPV, fL	5.46 ^ab^	5.49 ^a^	5.45 ^ab^	5.45 ^ab^	5.43 ^b^	0.010	0.006	0.018	0.006	0.289	0.925

^1^ Ctrl: without supplement; 0.5% humic acid; 1% humic acid; 1.5% humic acid; 2% humic acid. ^2^ Prepartum and postpartum data represent −60 to −4 days before and 1 to 2 days after calving, respectively. ^3^ MCV: mean corpuscular volume; MCH: mean corpuscular hemoglobin; MCHC: mean corpuscular hemoglobin concentration; MPV: mean platelet volume; TLC: total leukocyte count. ^a,b^ Values with different superscripts in the same row differ significantly (*p* ≤ 0.05).

**Table 6:** Effects of humic acid supplementation ^1^ on the serum biochemical parameters of cows during the pre- and postpartum periods ^2^ and calving day.

Item ^3^	Treatment					SEM	*p*-Values				
	Control	0.5% Humic Acid	1% Humic Acid	1.5% Humic Acid	2% Humic Acid		Treat	Linear	Quadratic	Time	Treat × Time
Prepartum											
Glucose, mg/dL	60.0	60.0	59.8	59.5	60.6	0.486	0.576	0.513	0.733	<0.001	0.651
ALT, U/L	18.9	19.1	19.2	19.0	19.3	0.137	0.230	0.508	0.356	<0.001	0.828
AST, U/L	82.7	81.3	81.2	81.7	80.7	0.556	0.108	0.152	0.180	<0.001	0.907
ALP, U/dL	96.9	96.7	96.1	96.2	96.0	0.564	0.813	0.898	0.261	<0.001	0.620
GGT, U/dL	23.2	23.0	23.2	22.9	23.1	0.145	0.350	0.455	0.517	<0.001	0.083
Triglyceride, mg/dL	14.7	14.4	15.2	14.6	15.1	0.346	0.497	0.640	0.413	0.003	0.515
Cholesterol, mg/dL	131	127	133	128	126	4.297	0.702	0.992	0.941	<0.001	0.688
NEFAs, mmol/L	0.168	0.161	0.157	0.159	0.165	0.0032	0.175	0.049	0.211	<0.001	<0.001
BHBA, mmol/L	0.26	0.25	0.24	0.25	0.25	0.003	0.582	0.887	0.237	<0.001	0.497
IgG, mg/mL	29.5 ^b^	30.1 ^a^	30.2 ^a^	29.9 ^ab^	29.6 ^b^	0.103	<0.001	<0.001	0.332	<0.001	0.007
Calving day											
Glucose, mg/dL	52.5	51.1	52.0	53.8	50.4	1.508	0.569	0.797	0.651	-	-
ALT, U/L	22.2	23.1	22.6	23.0	22.3	0.554	0.712	0.263	0.906	-	-
AST, U/L	94.3	91.0	91.7	93.4	96.5	1.938	0.293	0.067	0.562	-	-
ALP, U/dL	106	103	105	105	104	1.466	0.842	0.484	0.960	-	-
GGT, U/dL	25.1	24.7	25.5	24.6	24.1	0.439	0.257	0.727	0.665	-	-
Triglyceride, mg/dL	16.9	17.0	17.0	15.3	17.5	1.119	0.686	0.923	0.523		
Cholesterol, mg/dL	177	200	182	142	201	20.9	0.267	0.671	0.269	-	-
NEFAs, mmol/L	0.88 ^a^	0.73 ^b^	0.74 ^b^	0.90 ^a^	0.89 ^a^	0.053	0.037	0.008	0.269	-	-
BHBA, mmol/L	0.87	0.91	0.80	0.85	0.95	0.063	0.540	0.799	0.668	-	-
IgG, mg/mL	28.4^b^	30.8^a^	31.0^a^	29.3^ab^	28.5^b^	0.432	<0.001	<0.001	0.903	-	-
Postpartum											
Glucose, mg/dL	50.4	52.7	52.2	54.1	50.7	0.990	0.061	0.060	0.169	0.565	0.263
ALT, U/L	22.5	21.5	22.4	22.4	22.6	0.344	0.154	0.057	0.149	0.662	0.417
AST, U/L	90.6	90.9	92.8	93.7	92.6	1.500	0.556	0.860	0.089	0.885	0.799
ALP, U/dL	105	106	104	103	108	1.148	0.132	0.642	0.400	0.198	0.130
GGT, U/dL	25.1	25.5	25.4	24.9	24.9	0.347	0.686	0.286	0.394	0.109	0.688
Triglyceride, mg/dL	17.2 ^ab^	16.5 ^ab^	17.4 ^ab^	13.9 ^b^	17.7 ^a^	0.726	0.003	0.511	0.093	0.533	0.045
Cholesterol, mg/dL	217	188	198	176	188	21.2	0.722	0.478	0.371	0.579	0.065
NEFAs, mmol/L	0.90	0.80	0.83	0.82	0.88	0.036	0.370	0.054	0.817	0.505	0.538
BHBA, mmol/L	0.82 ^ab^	0.75 ^b^	0.76 ^b^	0.81 ^ab^	0.94 ^a^	0.047	0.040	0.043	0.238	0.896	0.183
IgG, mg/mL	29.8 ^a^	30.2 ^a^	29.6 ^ab^	29.7 ^ab^	28.6 ^b^	0.259	0.001	0.060	0.011	0.565	0.172

^1^ Ctrl: without supplement; 0.5% humic acid; 1% humic acid; 1.5% humic acid; 2% humic acid. ^2^ Prepartum and postpartum data represent −60 to −4 days before and 1 to 2 days after calving, respectively. ^3^ AST: aspartate aminotransferase; ALT: alanine aminotransferase; ALP: alkaline phosphatase; GGT: gamma glutamyl transferase; high-density lipoproteins; low-density lipoproteins; NEFAs: non-esterified fatty acids; BHBA: beta hydroxybutyric acid; IgG: immunoglobulin G. ^a,b^ Values with different superscripts in the same row differ significantly (*p* ≤ 0.05).

**Table 7:** Effects of humic acid supplementation ^1^ to dairy cows during dry period on serum physiological parameters of calves.

Item ^2^	Treatment					SEM	*p*-Value		
	Control	0.5% Humic Acid	1% Humic Acid	1.5% Humic Acid	2% Humic Acid		Treat	Linear	Quadratic
Calving day									
TLC, 10^9^/ml	9.12	8.94	9.00	9.06	9.23	0.153	0.719	0.222	0.692
Lymphocytes, %	3.50	3.46	3.54	3.47	3.57	0.068	0.749	0.560	0.659
Neutrophil, %	3.70	3.66	3.66	3.71	3.70	0.040	0.809	0.344	0.671
Monocytes, %	0.34	0.35	0.34	0.33	0.35	0.006	0.338	0.385	0.373
RBC, 10^6^/dL	6.92	6.97	6.79	7.03	7.10	0.142	0.614	0.638	0.764
Hemoglobin, g/dL	9.95	10.2	10.0	10.4	10.1	0.297	0.809	0.778	0.429
Hematocrit, %	31.5	31.6	31.3	31.4	31.4	0.232	0.894	0.960	0.374
MCV, fL	34.9	34.8	34.7	34.6	35.4	0.421	0.695	0.496	0.987
MCH, pg	12.4	11.6	12.1	12.2	12.2	0.309	0.534	0.118	0.569
MCHC, g/dL	32.9	33.3	32.8	33.0	32.6	0.455	0.912	0.623	0.605
Platelets, 10/L	252	251	252	250	252	0.642	0.388	0.420	0.957
Cholesterol, mg/dL	159 ^ab^	166 ^ab^	151 ^ab^	145 ^b^	180 ^a^	7.80	0.021	0.581	0.319
MPV, fL	5.61	5.64	5.66	5.64	5.63	0.032	0.899	0.428	0.576
IgG, mg/mL	0.49	0.51	0.49	0.50	0.50	0.014	0.909	0.854	0.637
Day 1									
TLC, 10^9^/ml	9.08	9.02	8.93	8.97	38.96	0.143	0.952	0.762	0.471
Lymphocytes, %	3.52	3.38	3.51	3.50	3.45	0.063	0.493	0.256	0.488
Neutrophil, %	3.65	3.74	3.73	3.71	3.72	0.037	0.505	0.105	0.611
Monocytes, %	0.34	0.32	0.33	0.34	0.34	0.005	0.287	0.045	0.623
RBC, 10^6^/dL	7.11	6.93	6.83	7.04	6.69	0.149	0.295	0.636	0.373
Hemoglobin, g/dL	9.97	9.68	9.92	9.75	9.98	0.278	0.911	0.454	0.923
Hematocrit, %	31.4	31.2	31.6	31.9	31.6	0.221	0.172	0.389	0.030
MCV, fL	34.9 ^ab^	35.7 ^a^	35.5 ^ab^	33.9 ^b^	34.6 ^ab^	0.397	0.018	0.041	0.043
MCH, pg	12.4 ^a^	12.0 ^ab^	11.3 ^ab^	11.2 ^b^	12.0 ^ab^	0.301	0.040	0.115	0.011
MCHC, g/dL	33.5	32.8	32.5	32.6	32.8	0.476	0.603	0.289	0.226
Platelets, 10/L	251	250	252	250	251	0.699	0.462	0.968	0.322
Cholesterol, mg/dL	163 ^b^	162 ^b^	147 ^bc^	129 ^c^	178 ^a^	7.32	<0.001	0.189	0.018
MPV, fL	5.67 ^a^	5.68 ^a^	5.65 ^ab^	5.55 ^b^	5.66 ^ab^	0.029	0.027	0.895	0.016
IgG, mg/mL	31.9 ^ab^	33.5 ^a^	32.9 ^ab^	32.6 ^ab^	31.4 ^b^	0.510	0.045	0.006	0.419
Day 2									
TLC, 10^9^/ml	9.15	8.91	9.01	9.06	9.02	0.140	0.777	0.231	0.947
Lymphocytes, %	3.47	3.50	3.59	3.51	3.56	0.072	0.773	0.746	0.354
Neutrophil, %	3.72	3.64	3.69	3.69	3.64	0.036	0.567	0.404	0.980
Monocytes, %	0.33	0.34	0.34	0.35	0.33	0.006	0.315	0.503	0.441
RBC, 10^6^/dL	6.80	6.87	6.80	6.85	7.13	0.145	0.365	0.664	0.588
Hemoglobin, g/dL	9.70	9.67	9.35	10.3	10.1	0.275	0.130	0.371	0.213
Hematocrit, %	31.5	31.4	31.5	31.6	31.7	0.237	0.936	0.467	0.642
MCV, fL	35.0	34.7	35.4	34.8	35.4	0.427	0.682	0.664	0.506
MCH, pg	11.5	12.1	11.6	12.2	12.3	0.289	0.145	0.583	0.365
MCHC, g/dL	33.4	33.7	33.6	32.6	32.8	0.447	0.344	0.397	0.138
Platelets, 10/L	251	251	252	252	250	0.628	0.277	0.356	0.615
Cholesterol, mg/dL	160 ^b^	158 ^b^	146 ^bc^	127 ^c^	175 ^a^	7.08	<0.001	0.131	0.021
MPV, fL	5.68	5.61	5.68	5.64	5.66	0.032	0.462	0.215	0.875
IgG, mg/mL	32.1 ^b^	33.2 ^ab^	34.1 ^a^	32.7 ^b^	32.2 ^b^	0.451	0.020s	0.007	0.501

^1^ Ctrl: without supplement; 0.5% humic acid; 1% humic acid; 1.5% humic acid; 2% humic acid. ^2^ MCV: mean corpuscular volume; MCH: mean corpuscular hemoglobin; MCHC: mean corpuscular hemoglobin concentration; MPV: mean platelet volume; TLC: total leukocyte count; IgG: immunoglobulin G. ^a,b,c^ Values with different superscripts in the same column are significantly different (*p* ≤ 0.05).

**Table 8:** Effects of humic acid supplementation ^1^ to dairy cows during dry period on serum biochemical ^2^ parameters of calves.

Item ^2^	Treatment					SEM	*p*-Value		
	Control	0.5% Humic Acid	1% Humic Acid	1.5% Humic Acid	2% Humic Acid		Treat	Linear	Quadratic
Calving day									
ALT, U/L	13.5	13.9	13.9	13.5	13.7	0.205	0.415	0.107	0.725
AST, U/L	47.8	49.0	48.3	49.6	48.9	0.531	0.141	0.291	0.166
ALP, U/dL	124	123	124	123	125	0.851	0.643	0.358	0.836
GGT, U/dL	79.3	78.1	74.2	77.9	77.9	2.023	0.449	0.331	0.366
Day 1									
ALT, U/L	13.6	13.8	13.4	13.6	13.4	0.216	0.724	0.520	0.368
AST, U/L	49.1	48.0	48.8	48.9	48.1	0.507	0.419	0.374	0.774
ALP, U/dL	326	330	333	326	325	8.587	0.950	0.525	0.956
GGT, U/dL	292	296	287	288	290	4.41	0.687	0.739	0.194
Day 2									
ALT, U/L	13.6	13.4	13.5	13.7	13.6	0.220	0.950	0.645	0.583
AST, U/L	48.5	48.6	48.2	48.4	48.3	0.501	0.992	0.983	0.705
ALP, U/dL	337	328	311	324	316	8.44	0.232	0.412	0.083
GGT, U/dL	415	412	425	425	422	7.23	0.536	0.921	0.091

^1^ Ctrl: without supplement; 0.5% humic acid; 1% humic acid; 1.5% humic acid; 2% humic acid. ^2^ AST: aspartate aminotransferase; ALT: alanine aminotransferase; ALP: alkaline phosphatase; GGT: gamma glutamyl transferase.

**Table 9:** Effects of humic acid supplementation ^1^ during the dry period on the BCS of dairy cows.

BCS					
	**−60**	−30	Calving Day	15	60
Ctrl	3.60 ± 0.70	3.60 ± 0.15	3.70 ± 0.15	3.15 ± 0.20	3.00 ± 0.15
0.5	3.60 ± 0.10	3.70 ± 0.15	3.60 ± 0.10	3.00 ± 0.15	3.00 ± 0.15
1	3.60 ± 0.15	3.70 ± 0.20	3.75 ± 0.15	3.20 ± 0.15	2.90 ± 0.10
1.5	3.60 ± 0.20	3.50 ± 0.15	3.75 ± 0.10	3.00 ± 0.15	3.05 ± 0.10
2	3.80 ± 0.15	3.55 ± 0.25	3.60 ± 0.20	3.00 ± 0.10	3.10 ± 0.10
*p*-Value	0.517	0.538	0.784	0.952	0.924

^1^ Ctrl: without supplement; 0.5% humic acid; 1% humic acid; 1.5% humic acid; 2% humic acid.

## Data Availability

The original contributions presented in this study are included in the article.
